# Association Study of Mannose-Binding Lectin Levels and Genetic Variants in Lectin Pathway Proteins with Susceptibility to Age-Related Macular Degeneration: A Case-Control Study

**DOI:** 10.1371/journal.pone.0134107

**Published:** 2015-07-24

**Authors:** Michael Osthoff, Melinda M. Dean, Paul N. Baird, Andrea J. Richardson, Mark Daniell, Robyn H. Guymer, Damon P. Eisen

**Affiliations:** 1 Victorian Infectious Diseases Service at the Doherty Institute, Melbourne Health, Melbourne, Victoria, Australia; 2 Department of Medicine, Royal Melbourne Hospital, University of Melbourne, Melbourne, Victoria, Australia; 3 Department of Infectious Diseases and Hospital Epidemiology, University Hospital Basel, Basel, Switzerland; 4 Research and Development, Australian Red Cross Blood Service, Kelvin Grove, Queensland, Australia; 5 Centre for Eye Research Australia, University of Melbourne, Royal Victorian Eye and Ear Hospital, East Melbourne, Victoria, Australia; University of Dundee, UNITED KINGDOM

## Abstract

**Background:**

In age-related macular degeneration (AMD) the complement system is thought to be activated by chronic oxidative damage with genetic variants identified in the alternative pathway as susceptibility factors. However, the involvement of the lectin pathway of complement, a key mediator of oxidative damage, is controversial. This study investigated whether mannose-binding lectin (MBL) levels and genetic variants in lectin pathway proteins, are associated with the predisposition to and severity of AMD.

**Methods:**

MBL levels and single nucleotide polymorphisms (SNPs) in the *MBL2* and the ficolin-2 (*FCN2*) gene were determined in 109 patients with AMD and 109 age- and sex-matched controls.

**Results:**

MBL expression levels were equally distributed in both cases (early and late AMD) and controls (p>0.05). However, there was a trend towards higher median MBL levels in cases with late AMD compared to cases with early AMD (1.0 vs. 0.4 μg/ml, p = 0.09) and MBL deficiency (<0.5 μg/ml) was encountered less frequently in the late AMD group (35% vs 56%, p = 0.03). *FCN2* and *MBL2* allele frequencies were similarly distributed in early and late AMD cases compared with controls (p>0.05 for all analyses) as were *MBL2* genotypes. Similarly, there was no significant difference in allele frequencies in any SNPs in either the *MBL2* or *FCN2* gene in cases with early vs. late AMD.

**Conclusions:**

SNPs of lectin pathway proteins investigated in this study were not associated with AMD or AMD severity. However, MBL levels deserve further study in a larger cohort of early vs. late AMD patients to elucidate any real effect on AMD severity.

## Introduction

Age-related macular degeneration (AMD) is the leading cause of progressive loss of central vision and ultimately severe vision impairment in the elderly population of developed countries. With the overall ageing of the population, its prevalence is expected to increase by at least 50% in the next 10 years [1]. Approximately 30% of persons over 75 years of age have some signs of AMD, and about 6% of persons in this age group suffer from the most advanced forms of AMD [2]. The early stage of the disease is characterized by a thickening of Bruch’s membrane and accumulation of deposits in the central retina (macula), beneath the retinal pigment epithelium (RPE), called drusen. Ongoing deposition and subsequent inflammation result in two advanced forms: geographic atrophy (dry AMD) with loss of RPE and photoreceptors, and choroidal neovascularisation (wet AMD) with leaking choroidal vessels invading the retina, both leading to an irreversible loss of central vision without treatment [3].

Both genetic and environmental factors (e.g. smoking and diet) have been implicated in this complex disease (reviewed in [4]). Regarding genetics, dysregulated local complement activation and subsequent chronic inflammation are considered to play an essential role in the pathogenesis of AMD (reviewed in [2]). Indeed, genome-wide and targeted linkage studies have identified several components of the complement system as major susceptibility loci for the development of AMD [5–7]. Besides a strong genetic association of mutations in the regulatory/inhibitory proteins of the alternative pathway (complement factor H and B, and Factor H-like protein) with AMD there is also evidence demonstrating an involvement of several other components of the complement system (e.g. C2, C3, complement factor H related proteins), that either intensify complement attacks, slow complement activation, or alter other control proteins, in addition to molecules from the cholesterol and angiogenesis pathway [4,8]. Hence, the genetic complexity of AMD is much greater than initially thought. Additionally, certain pathogens (like *Chlamydia pneumoniae* or *Cytomegalovirus*) have been implicated in the pathogenesis of AMD (by inducing a chronic inflammatory state) [9,10] and in AMD disease progression when in the presence of the CC risk genotype of the single nucleotide polymorphism (SNP) rs1061170 (Y402H) [11].

Although the current understanding of the pathogenesis of AMD is that chronic oxidative damage over time perpetuates a chronic inflammatory state via activation of the complement cascade, it remains to be determined which tissue ligands trigger complement activation and which components of the complement system are actually responsible for the damage.

Activation of the complement cascade is possible via one of three pathways, the classical, the lectin, and the alternative pathway, the latter one having a crucial role in amplifying complement activation. The lectin pathway is activated after binding of mannose-binding lectin (MBL) or ficolins (ficolin-1, -2, and -3) to pathogens or dying cells with subsequent activation of MBL-associated serine protease-1 and -2 and assembly of the C3 convertase [12]. Inter-individual serum concentrations of these pattern recognition receptors vary to a certain degree with MBL showing the greatest range (from undetectable to about 10 ug/ml) [13]. Well characterized polymorphisms in the exon and promoter region of the *MBL2* gene on chromosome 10 are responsible for the remarkable variations in serum MBL concentration in healthy individuals [14]. Notably, almost a third of the population worldwide display moderate to severe MBL deficiency[14]. Similarly, a number of major polymorphisms have been described for the ficolin-2 gene (*FCN2*) with a much weaker genotypic/phenotypic relationship than is the case for MBL [15]. Both MBL and ficolins have been shown to be involved in the binding and removal of certain pathogens and dying cells leading to complement activation in an antibody- and complement C1q independent manner [16–19]. Hence, MBL deficiency and/or low ficolin levels might lead to an impaired clearance of apoptotic debris or certain pathogens in AMD and consequently to sustained inflammation with activation of other complement pathways. However, a recent study has identified an antithetical involvement of the lectin pathway in AMD. In an *in vitro* model of oxidatively stressed RPE cells, the lectin pathway was found to be crucial in triggering activation of the complement cascade after binding to natural IgM antibodies bound to neoepitopes with subsequent amplification via the alternative pathway, whereas the classical pathways was not involved [20]. Comparable data in human AMD are scarce and essentially limited to a single study by Dinu V. et al., which identified a single nucleotide polymorphisms in the *MBL2* gene locus in a focused, pathway-based (instead of a genome-wide) association study, that was associated with an increased risk to develop wet AMD[21]. Given the potential involvement of the lectin pathway in the pathogenesis of AMD and the limited evidence from previous human studies we investigated the role of two lectin pathway pattern recognition receptors, MBL and ficolin-2, in the predisposition to AMD in a case-control study.

## Patients and Methods

### Ethics Statement

This matched case-control study was approved by both the Melbourne Health and the Royal Victorian Eye and Ear Hospital Human Research and Ethics Committees and all participants gave written informed consent for the study.

### Participants

136 individuals with AMD were recruited at the Centre for Eye Research Australia and 132 individuals with a similar age range but without evidence of AMD were recruited either at the Centre for Eye Research Australia or at the Royal Melbourne Hospital cataract surgery clinics. Subsequently, AMD cases and controls were matched for age (within 5 years) and sex, yielding a cohort of 109 participants in each group for primary analyses.

### AMD detection and definition

Digital non-stereoscopic fundus photography of the macular and optic disc was performed and graded in all subjects as described previously [22]. Participants were categorized on the status of the worse affected eye. Controls demonstrated bilateral normal fundi without evidence of drusen of any size. Exclusion criteria for both groups included: glaucoma, significant cataracts, any corneal pathology that could compromise vision, diabetes, uncontrolled hypertension, amblyopia, neurological or systemic disease affecting vision or any medication known to affect the retina. All cases with the early stages of AMD had intermediate AMD with the presence of drusen > 125 μm, with or without the presence of pigmentary abnormalities. Evidence of choroidal neovascularization (CNV), geographic atrophy or a disciform scar qualified for a diagnosis of late AMD [23].

### Definition of endpoints

The main aim of this exploratory study was to compare the frequency of MBL deficiency (defined as serum MBL levels < 0.5 μg/ml) in cases and controls [24]. As genetic material was available in the same subjects associations of *MBL2* and *FCN2* SNPs with AMD susceptibility and severity were investigated.

### Determination of MBL plasma levels

Quantification of serum MBL levels was performed by an investigator blinded to any patient data using a mannan-binding enzyme-linked immunosorbent assay as previously described [25]. Briefly, mannan-coated microtitre plates were incubated with samples at 1:25 and 1:100 dilutions for 90 min at room temperature followed by detection of bound MBL with a biotinylated monoclonal anti-MBL antibody (HYB 131–01, BioPorto Diagnostics, Denmark). After incubation with ExtrAvidin peroxidase conjugate (Sigma-Aldrich, Australia), plates were developed with TMB substrate solutions (BD OptEIA, BD Biosciences, Australia), stopped with 1 M H_2_SO_4_ (Sigma-Aldrich, Australia) and read immediately on a Bio-Rad plate reader (Bio-Rad, Australia). MBL levels were calculated against a standard pool serum (BioPorto Diagnostics, Denmark). MBL deficiency was defined as serum level < 0.5 μg/ml and, severe as < 0.1 μg/ml, respectively [26].

### *MBL2* and ficolin-2 genotyping

*MBL2* and *FCN2* promoter and exon SNPs were determined by allele specific polymerase chain reaction (PCR) using TaqMan fluorescent probes (TaqMan genotyping assays, Life Technologies, Australia). For assay details, see S1 Table. Stored genomic DNA was used for all cases and all controls recruited at the Centre for Eye Research Australia. For controls recruited at the Royal Melbourne Hospital, DNA lysates were prepared from 2μl of stored buffy coat according to the manufacturer’s instruction (TaqMan Sample-to-SNP, Life Technologies, Australia), and stored genomic DNA was used for all cases. For all TaqMan assays, DNA amplification was carried out in 5μL volume reactions containing 1μl of DNA lysate or 20ng of genomic DNA, 0.25μl TaqMan genotyping assay mix, 2.5μl TaqMan GTXpress Master Mix (Life Technologies, Australia) and 1.25μl DNase-free water. All reactions were performed in 384-well plates and in the ViiA 7 thermocycler (Life Technologies, Australia) according to the manufacturer’s instructions. For allelic discrimination end-point fluorescence was read at 25°C, and the ViiA 7 software was used to analyze the results (Life Technologies, Australia).

*MBL2* SNPs were chosen on the basis of their remarkable impact on MBL levels compared to other promoter or exon polymorphisms. *MBL2* genotypes were classified as low (XA/YO, YO/YO), intermediate (XA/XA, YA/YO) or high (YA/YA, XA/YA) producing genotypes according to published literature [24] with exon 1 variant alleles (rs1800450 (codon 54), rs1800451 (codon 57) or rs5030737 (codon 52)) collectively designated as O and the wild-type allele as A, and the promoter variant and the wild-type allele (rs7096206 (-221 X/Y)) designated as X and Y, respectively. Common *FCN2* promoter and exon SNPs were analyzed separately and combined as haplotype [27].

### Statistical analysis

This study sample was powered to detect an increase in the frequency of MBL deficiency (defined as MBL serum levels < 0.5 μg/ml) by a factor of 2.3 in AMD cases similar to the study of C3 polymorphisms and AMD susceptibility [28] with an expected frequency of MBL deficiency in the control population of 25% at the 5% level of significance.

To investigate MBL and ficolin-2 as potential risk factors for AMD, matched univariate analysis was performed by running conditional logistic regression on one variable at a time with AMD as the dependent variable. In addition, Wilcoxon signed-rank test was applied to compare MBL levels in cases and matched controls. Haplotype frequencies were analyzed by expectation-maximum algorithm.

We used Pearson's chi-square test for comparisons of categorical variables and allele and genotype frequencies and to check for Hardy–Weinberg equilibrium. For AMD severity analysis MBL levels were analyzed using the Mann-Whitney-U-Test. All testing was two-tailed. Haplotype and linkage disequilibrium analysis was carried out with the Haploview program (version 4.2). All other analyses were performed using SPSS statistics, version 17.0 (SPSS Inc., USA).

## Results

### Baseline characteristics

136 cases and 132 controls were genotyped for the *MBL* and *FCN2* genes. After matching for age and gender, the final study population consisted of 109 cases and controls. The mean age (standard deviation) was 76.2 (8.7) years in cases and 75.0 (8.1) in controls with 62.4% being female in both groups. Cases comprised 34 (31%) patients with neovascular AMD in their worst eye, 20 (18%) patients with geographic atrophy in their worst eye, and 55 (51%) patients with signs of the early stages of AMD had intermediate AMD in their worst eye. Two individuals did not have a final grading but were deemed to have AMD at some level. Controls had no signs of AMD on retinal photography.

Cases and controls were all successfully genotyped at 4 loci in the *MBL2* gene and 6 loci in the *FCN2* gene, and allele frequencies at all positions were in agreement with the predicted Hardy-Weinberg equilibrium (S1 Table) (p>0.05). As expected median, MBL levels significantly correlated with *MBL2* genotypes (<0.001 (interquartile range (IQR) 0–0.001) for low (XA/YO, YO/YO), 0.3 (IQR 0.2–0.8) for intermediate (XA/XA, YA/YO), and 1.6 (1.0–2.4) for high (YA/YA, XA/YA) producing *MBL2* genotypes, p<0.001).

### MBL and ficolin-2 and age-related macular degeneration

Median MBL levels were similar in cases and controls (0.7 (IQR 0.2–1.6) vs. 0.7 (IQR 0.1–1.7) μg/ml, respectively, p = 0.9) as was the frequency of moderate and severe MBL deficiency (46 vs. 43%, respectively, p = 0.7 and 19 vs. 23%, respectively, p = 0.5).

Frequencies of *MBL2* exon or promoter variant alleles and genotypes did not differ significantly between AMD cases (early and late) and controls (Table 1). Similarly, *FCN2* haplotypes and variant alleles were equally distributed with the exception of the variant haplotype AGAGTG (−986G> A, −602G>A, −557A>G, −4A>G +6359 C>T and +6424 G>T), which was less frequently observed in cases (Table 2). When cases with advanced AMD, i.e. neovascular or geographic atrophy, were grouped together and compared with controls, none of the loci were found to be significant at the p = 0.05 level (data not shown). Comparison of the intermediate AMD cases with controls also demonstrated that none of the loci at either gene were significant at the p = 0.05 level (data not shown).

**Table 1:** Analysis of MBL levels and *MBL2* polymorphisms in AMD cases and controls.

Variables	Cases	Controls	Univariate matched analysis	
	(n = 109)	(n = 109)	OR (95% CI)	P value
*MBL2* exon variants, n (%)				
rs1800451 (Codon 57)				
G/G	102 (94)	105 (96)	Reference	
G/A	7 (6)	5 (4)	1.75 (0.51–5.98)	0.37
A/A	-	-	-	-
rs1800450 (Codon 54)				
G/G	79 (72)	81 (74)	Reference	
G/A	28 (26)	25 (23)	1.12 (0.61–2.07)	0.7
A/A	2 (2)	3 (3)	0.72 (0.12–4.44)	0.7
rs5030737 (Codon 52)				
C/C	89 (82)	86 (79)	Reference	
C/T	19 (17)	20 (18)	0.93 (0.45–1.93)	0.9
T/T	1 (1)	3 (3)	0.33 (0.04–3.21)	0.3
*MBL2* promoter variant, n (%)				
rs7096206 (-221, Y/X)				
G/G	69 (63)	63 (58)	Reference	
G/C	37 (34)	40 (37)	0.84 (0.49–1.46)	0.5
C/C	3 (3)	6 (5)	0.46 (0.11–1.90)	0.3
*MBL2* genotypes, n (%)				
high producing	53 (49)	53 (49)	Reference	
intermediate producing	38 (35)	31 (28)	1.28 (0.63–2.60)	0.5
low producing	18 (16)	25 (23)	0.76 (0.37–1.58)	0.5
MBL levels (μg/ml), median (IRQ)	0.74 (0.17–1.56)	0.72 (0.12–1.69)	0.99 (0.78–1.27)	1.0
MBL <0.5μg/ml, n (%)	(46)	(43)	1.13 (0.65–1.98)	0.7
MBL <0.1 μg/ml, n (%)	(19)	(23)	0.81 (0.43–1.53)	0.5

*MBL2* genotypes were classified as low (XA/YO, YO/YO), intermediate (XA/XA, YA/YO) or high (YA/YA, XA/YA) producing genotypes with exon variant alleles (codon 57, 54 and 52) collectively designated as O and the wild-type allele as A, and the promoter variant and the wild-type allele designated as X and Y, respectively. Abbreviations: CI, confidence interval; MBL, mannose-binding lectin; OR, odds ratio; IQR, interquartile range; Y and A denote *MBL2* promoter and exon wildtype alleles, respectively.

**Table 2:** Analysis of *FCN2* polymorphisms in AMD cases and controls.

Variables	Cases	Controls	Univariate matched analysis	
	(n = 109)	(n = 109)	OR (95% CI)	P value
*FCN2–986*, n (%)				
G/G	26 (24)	23 (21)	Reference	
G/A	53 (49)	52 (48)	0.90 (0.44–1.83)	0.8
A/A	30 (27)	34 (31)	0.81 (0.40–1.63)	0.6
*FCN2–602*, n (%)				
G/G	63 (58)	65 (60)	Reference	
G/A	41 (38)	40 (37)	1.06 (0.61–1.86)	0.8
A/A	5 (4)	4 (3)	1.28 (0.34–4.89)	0.7
*FCN2–557*, n (%)				
A/A	83 (76)	82 (75)	Reference	
A/G	26 (24)	23 (21)	1.05 (0.56–1.97)	0.9
G/G^a^	0 (0)	4 (4)	0.11 (<0.01–1.05)	0.1
*FCN2–4*, n (%)				
A/A	63 (58)	53 (49)	Reference	
A/G	41 (38)	44 (40)	0.77 (0.43–1.36)	0.4
G/G	5 (4)	12 (11)	0.37 (0.13–1.09)	0.1
*FCN2 + 6359*, n (%)				
C/C	60 (55)	48 (44)	Reference	
C/T	44 (40)	48 (44)	0.72 (0.41–1.27)	0.3
T/T	5 (5)	13 (12)	0.32 (0.11–0.95)	**0.04**
*FCN2 + 6424*, n (%)				
G/G	88 (81)	83 (76)	Reference	
G/T	21 (19)	22 (20)	0.90 (0.48–1.70)	0.8
T/T^a^	0 (0)	4 (4)	0.10 (<0.01–1.01)	0.1
*Haplotypes (−986/−602/-557/−4/+6359/+6424)*, n (%)				
GGAACG	39 (36)	34 (31)	Reference	
AGAGTG	24 (22)	34 (31)	0.57 (0.35–0.93)	**0.02**
AAAACG	24 (22)	21 (19)	0.98 (0.59–1.63)	0.9
GGGACT	10 (9)	13 (12)	0.56 (0.30–1.05)	0.07
AGAACG	6 (6)	2 (2)	2.14 (0.68–6.73)	0.2

Abbreviations: CI, confidence interval; OR, odds ratio

^a^ Exact logistic regression was used to compute OR, CI and p value.

With regards to AMD severity there was no significant difference in *MBL2* allele and *FCN2* allele or haplotype frequencies in cases with early vs. late AMD (data not shown). However, there was a trend towards higher MBL levels in cases with late AMD compared to participants with early AMD changes (1.0 (IQR 0.3–1.9) vs. 0.4 (IQR 0.1–1.4) μg/ml, p = 0.09), and MBL deficiency (<0.5 μg/ml) was encountered less frequently in the late AMD group (19 (35%) vs 31 (56%), p = 0.03).

Overall, the above results were similar when analyzing the entire, unmatched cohort of 136 cases and 132 controls (data not shown).

## Discussion

The lectin pathway of complement has been implicated in the pathogenesis of AMD in several experimental models [20,29,30]. This is the first human study designed to examine the role of two lectin pathway pattern recognition molecules, MBL and ficolin-2, in the predisposition to and severity of AMD, exclusively.

Despite previous experimental studies that were the basis for our a priori hypotheses, we did not demonstrate any relevant associations of SNPs in the *MBL2* or *FCN2* gene or MBL protein levels with the early stages or late AMD in our matched case-control study. Similarly, *MBL2* and *FCN-2* polymorphisms had no influence on the severity of AMD in our cohort.

However, there was a non-significant trend towards higher MBL levels in cases with late AMD compared to early AMD corresponding to a significantly lower frequency of MBL deficiency.

This is in contrast to a complement pathway-focused association study by Dinu V et al., who identified a SNP in the intron region of the *MBL2* gene as being able to differentiate between early and late AMD [21]. The *MBL2* SNP identified in that study as being associated with AMD severity has neither been shown to influence serum MBL levels nor associate with other diseases such as colon cancer or breast cancer [31,32]. For these reasons we did not screen for this SNP in our cohort.

In addition, two experimental studies have demonstrated that the lectin pathway is at least partly responsible for complement activation in AMD *in vitro* and rodent models [20,29]. In the study by Joseph K et al., MBL and ficolins (mainly ficolin-1 and -3) were shown to be capable of activating the lectin pathway of complement after binding to natural antibodies attached to oxidatively stressed RPE cells with the alternative pathway acting as an amplification loop [20].

Several reasons might account for our inability to find an association of MBL deficiency and/or *MBL2*/*FCN2* polymorphisms in the present study. First, the lectin pathway, and MBL and ficolin-2 in particular, might indeed not be involved in the pathogenesis of AMD. This is supported by several genome-wide association studies that have not found a significant association in the region of those two genes [5,33,34]. Second, AMD is a complex disease with multiple environmental and genetic factors involved. Hence, previous *in vitro* and rodent models that have demonstrated a role of the lectin pathway in AMD, might not have been able to account for the complexity of human AMD. Third, our study was only powered to detect a 2.3-fold difference in the frequency of MBL deficiency defined as serum levels < 0.5 μg/ml (which was more frequent in controls than expected). Our negative result and insufficient power influenced our decision to not include other important genetic and environmental factors in our analysis for controls recruited at one site. This may have biased our results. SNPs involving the lectin pathway might still have some influence in AMD risk but at a lower frequency than that which we were powered to detect. It is still important to search for these rare haplotypes as they may provide important information about pathways and mechanisms involved in AMD pathogenesis [35],[36,37].

In this regard, a trend for higher MBL levels and a significantly lower frequency of MBL deficiency in the late AMD group is of interest. This finding was contrary to our hypothesis but might be worth exploring in larger cohorts as experimental studies have suggested that complement activation initiated by the lectin pathway potentiates retinal damage [20,29]. There is clearly plentiful evidence to suggest the involvement of the complement system in the pathogenesis of AMD. However, the exact mechanism of involvement remains to be elucidated.

Limitations of our study include the sample size, in particular of advanced cases of AMD and the absence of phenotypic ficolin-2 testing in our cohort. Although there is a significant correlation of *FCN2* genotypes with ficolin-2 serum levels, the ability of *FCN2* genotyping to predict serum concentrations is limited [15]. Hence, we cannot exclude an influence of ficolin-2 at the phenotypic level in AMD given that the variant *FCN2* haplotype AGAGTG was less common in AMD cases. In addition, we limited our analysis to two pattern recognition receptors of the lectin pathway. Ideally, future AMD association studies should include other important lectin pathway proteins like ficolin-1 and -3 and MASP-1 and -2. We were not able to match for important environmental risk factors like smoking in our study which might have biased our results. We were not able to provide data on the activation state of the complement system in cases and controls which could influenced MBL levels[38].

In conclusion, we were not able to find any evidence from our matched case-control study to support a relevant role for SNPs in either the *MBL2* or *FCN2* genes in the predisposition to and severity of AMD. We have not excluded a rarer association with AMD. Due to our observation of lower MBL levels in less severe AMD cases future studies are needed to validate this association and determine if indeed the lectin pathway of complement is ultimately involved in the pathogenesis of AMD.

## Supporting Information

S1 File Data file of matched case-control cohort. (XLSX)

S1 Table Taqman genotyping assay details including results of testing for Hardy-Weinberg equilibrium. (DOC)
