# Supplementary material for: Association Study of Mannose-Binding Lectin Levels and Genetic Variants in Lectin Pathway Proteins with Susceptibility to Age-Related Macular Degeneration: A Case-Control Study
Source: PLoS One. 2015 Jul 24;10(7):e0134107. doi: 10.1371/journal.pone.0134107 (PMC4514807; doi:10.1371/journal.pone.0134107)
Supplement: S1 Table — (DOC) [file pone.0134107.s002.doc]

Table S1: Taqman genotyping assay details (Life Technologies, Australia) including results of testing for Hardy-Weinberg equilibirum

| **Nucleotid Change** | **Localization** | **SNP Database ID** | **Assay Reference** | **Primers** | **Fluorescent Probes** | **HWE***  **p-value** |
| --- | --- | --- | --- | --- | --- | --- |
| *MBL2* -X/Y | 10q11.2-q21 | rs7096206 | C__27858274_10 |  |  | 0.22 |
| *MBL2* -B (codon 54) | 10q11.2-q21 | rs1800450 | C___2336609_20 |  |  | 1.0 |
| *MBL2* -C (codon 57) | 10q11.2-q21 | rs1800451 | C___2336608_20 |  |  | 0.84 |
| *MBL2 -*D (codon 52) | 10q11.2-q21 | rs5030737 | C___2336610_10 |  |  | 0.45 |
| *FCN2* -986 (G>A)[^1^](#_ENREF_1) | 9q34 | rs3124952 |  | 5‘-GGACCTCGGCATCCC-3‘ | VIC-ACCTGCCGCCATC | 0.89 |
|  |  |  |  | 5‘-CCACCACCGCACCCT-3‘ | FAM-CACCTGCTGCCATC |  |
| *FCN2 -602* (G>A) | 9q34 | rs3124953 | C__27461651_20 |  |  | 0.52 |
| *FCN2 -557* (A>G) | 9q34 | rs3811140 | C_____65537_10 |  |  | 1.0 |
| *FCN2 -4 (A>G)* | 9q34 | rs17514136 | C__25765134_10 |  |  | 1.0 |
| *FCN2 +6359 (C>T)* | 9q34 | rs17549193 | C__61859150_10 |  |  | 0.90 |
| *FCN2 +6424 (G>T)* | 9q34 | rs7851696 | C__29220549_20 |  |  | 0.91 |

Abbreviations: HWE, Hardy-Weinberg equilibrium; MBL, mannose-binding lectin; FCN2, ficolin-2

*The significance of deviations from Hardy-Weinberg equilibrium was tested using the Haploview program (version 4.2).

**Reference:**

1. Munthe-Fog L, Hummelshoj T, Hansen BE, et al. The impact of FCN2 polymorphisms and haplotypes on the Ficolin-2 serum levels*. Scand J Immunol*. Apr 2007;65(4):383-392.
